# Pain-related synaptic plasticity in spinal dorsal horn neurons: role of CGRP

**DOI:** 10.1186/1744-8069-2-31

**Published:** 2006-09-26

**Authors:** Gary C Bird, Jeong S Han, Yu Fu, Hita Adwanikar, William D Willis, Volker Neugebauer

**Affiliations:** 1 Department of Neuroscience and Cell Biology, The University of Texas Medical Branch, Galveston, Texas 77555-1069, USA

## Abstract

**Background:**

The synaptic and cellular mechanisms of pain-related central sensitization in the spinal cord are not fully understood yet. Calcitonin gene-related peptide (CGRP) has been identified as an important molecule in spinal nociceptive processing and ensuing behavioral responses, but its contribution to synaptic plasticity, cellular mechanisms and site of action in the spinal cord remain to be determined. Here we address the role of CGRP in synaptic plasticity in the spinal dorsal horn in a model of arthritic pain.

**Results:**

Whole-cell current- and voltage-clamp recordings were made from substantia gelatinosa (SG) neurons in spinal cord slices from control rats and arthritic rats (> 6 h postinjection of kaolin/carrageenan into the knee). Monosynaptic excitatory postsynaptic currents (EPSCs) were evoked by electrical stimulation of afferents in the dorsal root near the dorsal root entry zone. Neurons in slices from arthritic rats showed increased synaptic transmission and excitability compared to controls. A selective CGRP1 receptor antagonist (CGRP8-37) reversed synaptic plasticity in neurons from arthritic rats but had no significant effect on normal transmission. CGRP facilitated synaptic transmission in the arthritis pain model more strongly than under normal conditions where both facilitatory and inhibitory effects were observed. CGRP also increased neuronal excitability. Miniature EPSC analysis suggested a post- rather than pre-synaptic mechanism of CGRP action.

**Conclusion:**

This study is the first to show synaptic plasticity in the spinal dorsal horn in a model of arthritic pain that involves a postsynaptic action of CGRP on SG neurons.

## Background

Inflammatory processes in peripheral tissues lead to central sensitization in the spinal cord, which contributes to hyperalgesia and allodynia typically associated with inflammatory pain. Although evidence suggests that plastic changes in the spinal dorsal horn account for central sensitization, the relative contribution of pre- and postsynaptic mechanisms and of peripheral and supraspinal factors are not entirely clear. The superficial dorsal horn of the spinal cord, particularly substantia gelatinosa (SG), is a major projection site of small-diameter afferent nerve fibers that predominantly transmit nociceptive signals [1,2]. SG neurons also receive descending inputs from the brainstem [1,3]. Therefore, in addition to intraspinal neuroplastic changes, peripheral as well as supraspinal factors may contribute to central sensitization.

Pain-related neuroplastic changes in central nervous system (CNS) structures can be shown definitively by the electrophysiological analysis of synaptic transmission and neuronal excitability in spinal cord or brain slice preparations obtained from animals in which an experimental pain state has been induced [4-7]. The slice preparation allows the analysis of pain-related plasticity because it is disconnected from the site of peripheral injury (inflammation) and from other CNS areas, be it supraspinal sites (spinal cord slice) or spinal cord (brain slices). Therefore, changes measured in the slice preparation are maintained independently of continuous inputs to the area of interest. Accordingly, changes of synaptic circuitry in SG neurons were shown in slices from animals with complete Freund's adjuvant induced hindpaw inflammation [4,5,8,9] and synaptic plasticity was demonstrated in amygdala neurons from animals with knee joint arthritis [7,10,11].

The kaolin and carrageenan (K/C) induced knee joint arthritis is a well established model of inflammatory pain. Electrophysiological, pharmacological, neurochemical and behavioral studies have used this model to analyze pain mechanisms at different levels of the nervous system and showed the sensitization of primary afferent nerve fibers, spinal dorsal horn neurons and neurons in the central nucleus of the amygdala (CeA) [12-17]. Using slice preparations, synaptic plasticity was demonstrated in the CeA, but not yet in the spinal cord, in the K/C arthritis pain model.

The purpose of this study was to compare synaptic transmission and neuronal excitability in SG neurons in spinal cord slices from normal and from arthritic animals using patch-clamp recordings. Another goal was to determine the role of calcitonin gene-related peptide (CGRP) in pain-related spinal plasticity since CGRP has emerged as an important molecule at different levels of the pain neuraxis in the arthritis pain model.

CGRP is a 37 amino acid peptide that activates adenylyl cyclase and protein kinase A through G-protein-coupled receptors, including the CGRP1 receptor for which selective antagonists are available [18-21]. CGRP is involved in peripheral and spinal pain mechanisms [22-29]. We showed recently that CGRP also plays an important role in the transmission of nociceptive information to the amygdala through the spino-parabrachio-amygdaloid pathway [10].

The source of CGRP in the spinal cord dorsal horn is primary afferents. CGRP coexists with substance P in small-diameter afferent fibers, and CGRP containing terminals and CGRP receptors are found in the dorsal horn, including SG [30-33]. CGRP is released in the spinal dorsal horn by noxious stimulation and peripheral inflammation such as the K/C arthritis [26,34,35]. Peripheral inflammation also leads to changes in CGRP binding sites in the dorsal horn [32,36].

Spinal application of CGRP facilitates nociceptive behavior [24,37,38] and sensitizes the responses of dorsal horn neurons to innocuous and noxious peripheral stimulation [28,29,38,39] and to intraspinally administered excitatory amino acids [23] and substance P [39]. In a slice preparation, CGRP produced a slow depolarization and enhanced excitability of dorsal horn neurons; the effect on evoked synaptic transmission was not studied [40]. Conversely, block of spinal CGRP receptors with an antagonist (CGRP8-37) or antiserum induced antinociception in animal models of inflammatory [25,41-44] or central neuropathic pain [45]. CGRP8-37 also inhibited the responses of spinal dorsal horn neurons to transdermial electrical stimulation of the hindpaw [46] and to noxious mechanical stimulation of the knee joint [29]. CGRP8-37 prevented or reversed central sensitization of dorsal horn neurons in the arthritis and capsaicin pain models [28,29]. Arthritic CGRP knockout mice showed reduced nociceptive behavioral responses [47].

Although it is widely accepted that CGRP plays an important role in the modulation of spinal nociceptive processing, the cellular mechanisms and pre- or post-synaptic sites of action through which CGRP contributes to central sensitization remain to be determined. The present study addressed the role of CGRP in synaptic plasticity in the superficial dorsal horn *in vitro *in a model of arthritic pain induced *in vivo*. Our data show for the first time synaptic plasticity and increased excitability of SG neurons in the K/C arthritis pain model. A CGRP receptor antagonist inhibits synaptic plasticity whereas CGRP itself facilitates synaptic transmission through a postsynaptic mechanism that involves direct membrane effects on SG neurons.

## Results

Whole-cell patch-clamp recordings of SG neurons were made in spinal cord slices from normal naïve rats (n = 31 neurons) and rats with a knee joint arthritis induced 6 h before slices were obtained (n = 25 neurons). The recording sites were always visually verified to be in the central part of the gray translucent region forming lamina II. All SG neurons in this study showed monosynaptic responses (excitatory postsynaptic currents, EPSCs) to electrical stimulation of afferent fibers in the dorsal root (DR) near the dorsal root entry zone (DREZ). EPSCs were judged to be monosynaptic on the basis of stable latencies of the EPSC peak amplitude (coefficient of variation < 2%, [48,49]). Calculated from latency and distance between stimulation and recording sites, the conduction velocities (CV) ranged from 0.15 to 0.85 m/s (mean 0.46 ± 0.03 m/s), which is in the range of rodent C-fibers [48,49]. No difference in resting transmembrane potential (RMP) and input resistance (Ri) was detected between neurons from normal rats (RMP = -58.7 ± 1.7 mV; Ri = 211.8 ± 16.6 MΩ) and from arthritic rats (RMP = -57.0 ± 1.8 mV; Ri = 205.3 ± 13.7 MΩ).

### Synaptic plasticity in SG neurons in the arthritis pain model

**Figure 1:**
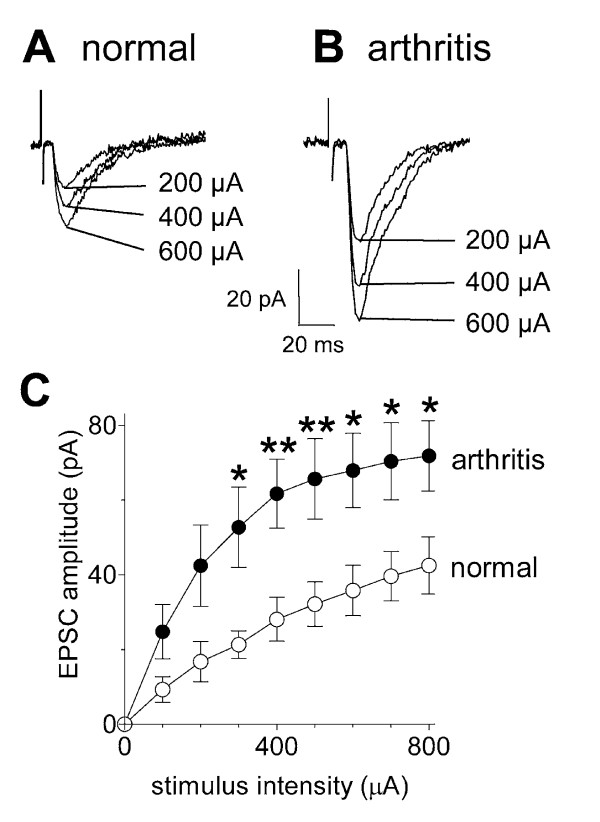
**Synaptic transmission in SG neurons is enhanced in the arthritis pain model**. **A,B**, Whole-cell voltage-clamp recordings of monosynaptic EPSCs evoked with increasing stimulus intensities in an SG neuron in a spinal cord slice from a normal animal and in an SG neuron in a slice from an arthritic animal (obtained 6 h post-induction of arthritis). Evoked monosynaptic EPSCs had larger amplitudes in arthritis than under control conditions. Square wave electrical stimuli of 150 μs duration were delivered at a frequency < 0.25 Hz. Stimulus intensity was increased from 0–800 μA. Each trace is the average of 3–4 EPSCs. Neurons were held at -60 mV. **C**, Input-output relationships of monosynaptic EPSC peak amplitudes (pA) evoked in SG neurons from normal rats (n = 16) and from arthritic rats (n = 9) were significantly different. * P < 0.05, ** P < 0.01 (two-way ANOVA followed by Bonferroni posttests). Data are given as the means ± SEM.

Input-output functions of monosynaptic inputs to SG neurons increased in the arthritis pain model (Figure 1). Monosynaptic EPSCs with progressively larger amplitudes were evoked by electrical DR/DREZ stimulation with increasing intensities. Compared with control SG neurons from normal animals, synaptic transmission was significantly enhanced in SG neurons recorded in slices from arthritic rats. Input-output relationships were obtained by measuring EPSC peak amplitude (pA) as a function of afferent fiber stimulus intensity (μA) for each neuron (see individual examples of an SG neuron in a slice from a normal animal [Fig. 1A] and in an SG neuron from an arthritic animal [Fig. 1B]). In arthritis, evoked monosynaptic EPSCs had larger amplitudes, but EPSC threshold was unchanged. The input-output relationships of SG neurons from control rats (n = 16) and SG neurons from arthritic rats (n = 9) were significantly different (Fig. 1C; P < 0.0001, F _1,207 _= 58.45, two-way ANOVA). These data show enhanced synaptic transmission at first-order synapses on SG neurons in the arthritis pain model. Enhanced synaptic transmission in the reduced slice preparation indicates synaptic plasticity because the arthritis pain-related changes are maintained, at least in part, independently of peripheral and supraspinal mechanisms.

### Increased excitability of SG neurons in the arthritis pain model

**Figure 2:**
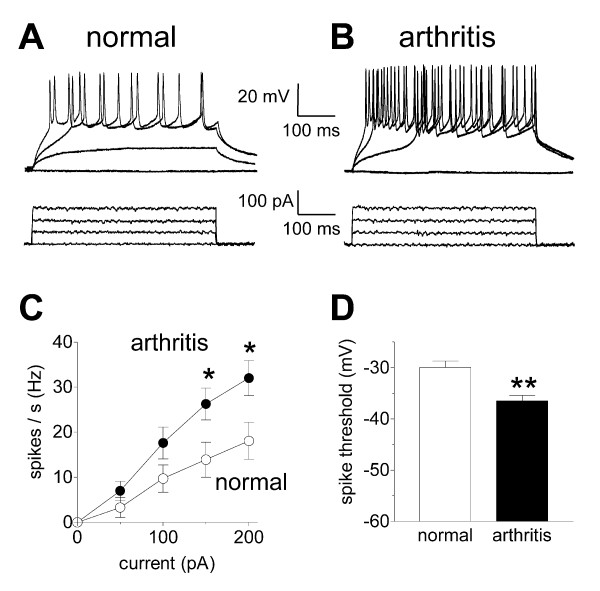
**Increased excitability of SG neurons in the arthritis pain model**. Increased action potential firing rates and decreased thresholds for action potentials were recorded in SG neurons in slices from arthritic rats compared to controls. **A, B**, Current-clamp recordings of action potentials (spikes) generated by direct intracellular injections of depolarizing current pulses of increasing magnitude (0 to 200 pA; 500 ms) in an SG neuron from a normal animal (A) and in an SG neuron from an arthritic animal (B). **C**, Analysis of the input-output relationships shows significantly increased spike frequency in arthritis (n = 13 neurons) compared to control (n = 25 neurons; P < 0.05; two-way ANOVA followed by Bonferroni posttests). **D**, Significantly decreased spike thresholds (membrane potentials at which action potential firing started) were recorded in SG neurons in arthritis (n = 11) compared to control neurons (n = 17; P < 0.01; unpaired t-test). * P < 0.05, ** P < 0.01.

Compared with control neurons, neurons from arthritic rats had a lower threshold and higher rate of action potential firing generated by direct depolarization of the cell via the recording electrode in current-clamp mode (Figure 2). Input-output functions of neuronal excitability were obtained by measuring the number of action potentials (Hz) evoked by depolarizing current pulses of increasing magnitude (0 to 200 pA; see individual examples in Fig. 2A and 2B). Input-output functions of SG neurons from arthritic animals (n = 13) were significantly increased compared to control neurons from normal animals (n = 25; Fig. 2C; P < 0.001; F _1,180 _= 12.77, two-way ANOVA). The threshold for evoking action potentials was lower in SG neurons from arthritic animals (n = 11) than in control neurons (n = 17), i.e., action potential firing occurred at more hyperpolarized membrane potentials (Fig. 2D; P < 0.01, unpaired t-test).

### Inhibition of pain-related synaptic plasticity by a CGRP1 receptor antagonist (CGRP8-37)

**Figure 3:**
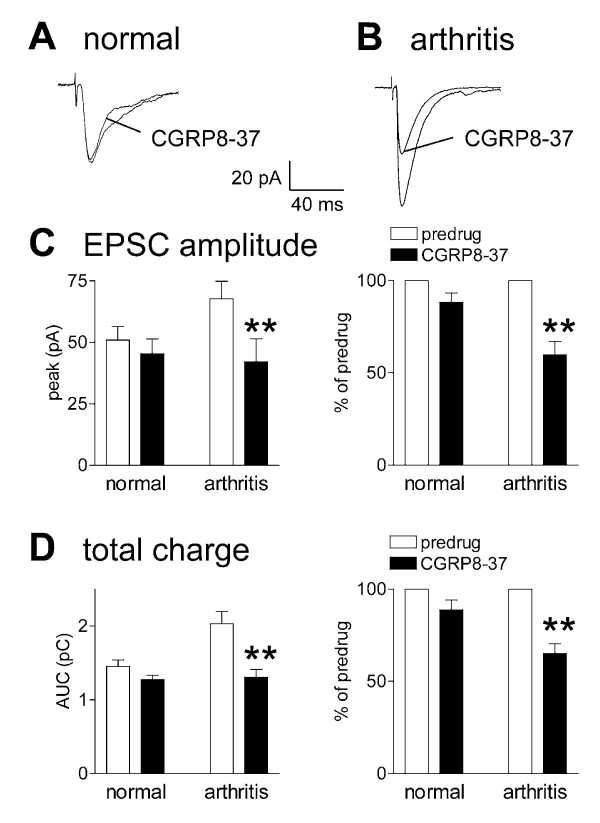
**CGRP8-37 inhibits pain-related synaptic plasticity but has no significant effect on normal synaptic transmission**. **A, B**, CGRP8-37 (1 μM) inhibited monosynaptic EPSCs recorded in an SG neuron in a slice from an arthritic rat (B) but not in another SG neuron in a slice from a normal rat (A). Each trace is the average of 8–10 monosynaptic EPSCs. **C, D**, CGRP8-37 (1 μM) significantly inhibited the EPSC peak amplitude (C), a measure of synaptic strength, and area under the curve (total charge, D) in SG neurons in slices from arthritic rats (P < 0.01, paired t-test, n = 5) but not in control neurons (n = 7) from normal rats. Analysis of raw data (pA, pC) is shown on the left; normalized data (% of predrug values) are shown on the right in C and D. Voltage-clamp recordings were made at -60 mV. CGRP8-37 was applied by superfusion of the slice in ACSF for 10–12 min. ** P < 0.01 (paired t-test).

CGRP8-37 (1 μM; 10 min) inhibited synaptic transmission in SG neurons in slices from arthritic animals but had no significant effect on normal synaptic transmission (Figure 3). Individual examples show that CGRP8-37 clearly inhibited monosynaptic EPSCs recorded in an SG neuron in a slice from an arthritic rat (Fig. 3B) but had little effect in an SG neuron in a slice from a normal rat (Fig. 3A). In the sample of SG neurons from arthritic rats (n = 5), CGRP8-37 inhibited synaptic strength (measured as peak amplitudes, Fig. 3C) and total charge (measured as area under the curve, Fig. 3D) significantly (P < 0.01, paired t-test), but had no significant effect on synaptic transmission in SG neurons from normal rats (n = 7). These data suggest that CGRP1 receptors are endogenously activated to facilitate synaptic transmission in the arthritic pain model. Next, we determined the effect and site of action of the receptor ligand CGRP.

### Synaptic facilitation by CGRP is enhanced in the arthritis pain model

**Figure 4:**
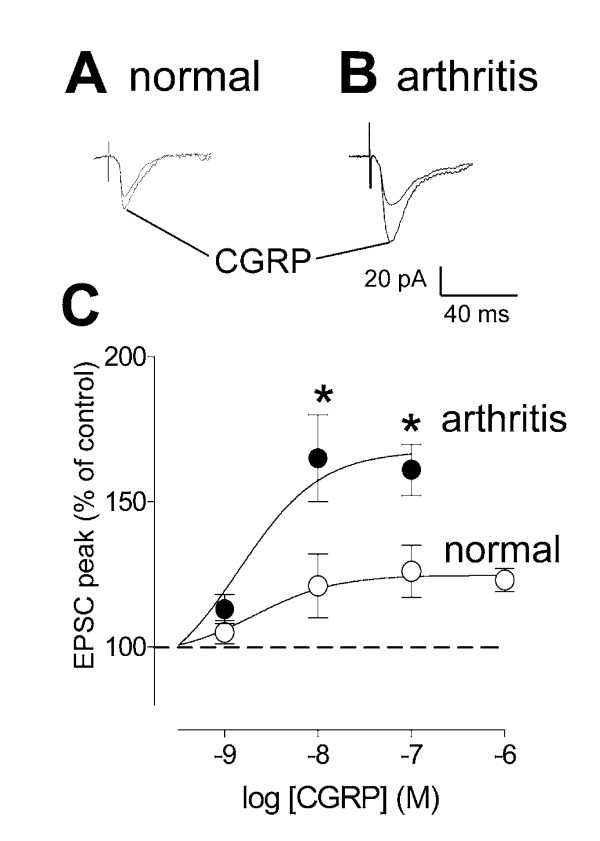
**Enhanced synaptic facilitation by CGRP in the arthritis pain model**. **A, B**, Whole-cell voltage-clamp recordings of monosynaptic EPSCs in an SG neuron in a slice from a normal animal (A) and in another SG neuron in a slice from an arthritic animal (B, 6 h postinduction of arthritis). CGRP (10 nM) potentiated synaptic transmission more strongly in arthritis than under normal conditions. Square wave electrical stimuli of 150 μs duration were delivered at a frequency < 0.25 Hz. Each trace is the average of 8–10 EPSCs. **C**, Concentration-response data show that the maximum effect (efficacy) of CGRP was significantly greater in SG neurons from arthritic rats (n = 16) compared to control neurons from normal animals (n = 10). Peak EPSC amplitudes during each concentration of CGRP were averaged and expressed as percent of predrug (baseline) control (100%). Sigmoid curves were fitted to the data using the following formula for nonlinear regression (GraphPad Prism 3.0; *Y *= *A*+(*B*-*A*)/[1+(10*C*/10*X*)*D*], where *A *= bottom plateau, *B *= top plateau, *C *= log(EC50), *D *= slope coefficient. Symbols show mean ± SEM. Neurons were held at -60 mV. CGRP was applied by superfusion of the slice in ACSF for 10 min. * P < 0.05 (two-way ANOVA followed by Bonferroni posttests).

CGRP enhanced synaptic transmission in a concentration-dependent fashion (Figure 4). Individual examples show that CGRP (10 nM; 10 min) increased monosynaptic EPSCs in an SG neuron from an arthritic rat (Fig. 4B) more strongly than in an SG neuron from a normal rat (Fig. 4A). Concentration-response data (Fig. 4C) show that the maximum facilitatory effect (efficacy) of CGRP was significantly (P < 0.01, F _1,26 _= 9.58, two-way ANOVA) greater in SG neurons from arthritic rats (n = 16) compared to control neurons from normal animals (n = 10). The potency of CGRP was comparable under normal conditions (EC_50 _= 2.0 nM) and in arthritis (EC_50 _= 1.4 nM). It should be noted that CGRP attenuated synaptic transmission in 6 of 16 SG neurons from normal animals but not in any SG neuron from arthritic animals.

### Postsynaptic effects of CGRP

**Figure 5:**
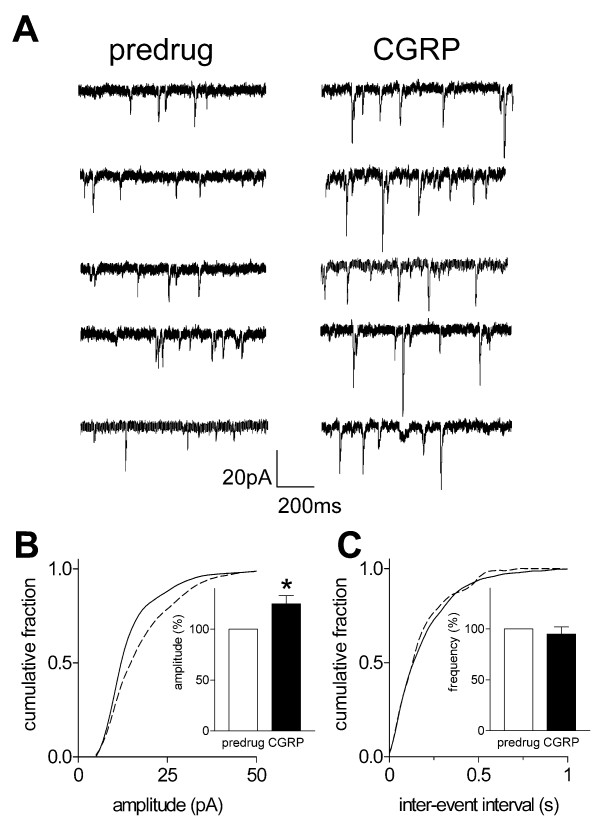
**Miniature EPSC (mEPSC) analysis indicates post- rather than pre-synaptic effects of CGRP**. **A**, Original current traces of mEPSC recorded in an individual SG neuron in the presence of TTX (1 μM) show that CGRP (10 nM; 10 min) increases amplitude but not frequency of mEPSCs. **B, C**: Normalized cumulative distribution analysis of mEPSC amplitude and frequency in the same neuron as in 5A shows that CGRP caused a significant shift toward higher amplitude (**B**, P < 0.001, Kolmogorov-Smirnov test) but had no effect on the interevent interval (frequency) distribution (**C**). In the sample of neurons (n = 5) CGRP selectively increased mean mEPSC amplitude (P < 0.05, paired t-test) but not mEPSC frequency (see bar histograms in **B, C**). Symbols and error bars represent mean ± SEM. Neurons were recorded in voltage-clamp at -60 mV. * P < 0.05.

**Figure 6:**
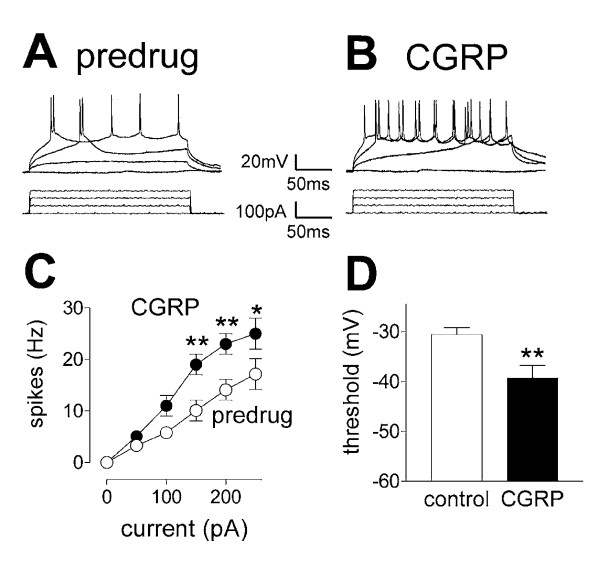
**CGRP increases neuronal excitability and induces direct membrane currents**. **A, B**, Current-clamp recordings of action potentials (spikes) generated in an SG neuron by direct intracellular injections of depolarizing current pulses of increasing magnitude (0 to 250 pA; 500 ms) before (A) and during CGRP (10 nM, B). **C**, CGRP increased input-output function by significantly increasing spike frequency (n = 5 neurons; P < 0.05–0.01, two-way ANOVA followed by Bonferroni posttests). For the measurement of action potential firing in current-clamp, neurons were recorded at -60 mV. **D**, CGRP (10 nM) also decreased spike thresholds (membrane potentials at which action potential firing started) significantly (n = 5; P < 0.01, paired t-test). * P < 0.05, ** P < 0.01.

To determine the site of action of CGRP we used well-established electrophysiological methods, including the analysis of miniature EPSCs (mEPSCs) (Figure 5) and neuronal excitability (Figure 6). Presynaptic changes at the transmitter release site affect mEPSC frequency, whereas changes at the postsynaptic membrane alter mEPSC amplitude (quantal size) [50,51]. CGRP (10 nM; 10 min) increased the amplitude of mEPSCs in TTX (1 μM)-containing ACSF without affecting their frequency, suggesting a post- rather than pre-synaptic site of action (Figure 5). This postsynaptic effect is illustrated in the current traces recorded in voltage-clamp mode in an individual SG neuron (Fig. 5A). Normalized cumulative distribution analysis of mEPSC amplitude and frequency shows that CGRP caused a significant shift toward higher amplitude in this neuron (see Fig. 5B and 5C) and also increased mean mEPSC amplitude in the sample of neurons (Fig. 5B, inset; P < 0.05, paired t-test, n = 5) but had no effect on the interevent interval (frequency) distribution (Fig. 5C, inset).

CGRP increased neuronal excitability (Figure 6). Action potentials were evoked in current-clamp mode by direct depolarizing current injections (500 ms) of increasing magnitude (0 to 250 pA) through the patch electrode (Fig. 6A and 6B). Input-output functions of neuronal excitability were obtained by averaging the frequency of action potentials (spikes) evoked at each current intensity (Fig. 6C). CGRP (10 nM; 10 min) increased the input-output function significantly (n = 5; P < 0.001; F _1,180 _= 12.77, two-way ANOVA) while lowering the threshold for action potential generation to more hyperpolarized membrane potentials (Fig. 6D; P < 0.01, paired t-test). In the presence of TTX (1 μM) CGRP (10 nM) also induced an inward membrane current that was significantly larger (P < 0.01, unpaired t-test) in SG neurons from arthritic rats (27.3 ± 3.1 pA, n = 5) than in SG neurons from normal rats (12.4 ± 2.9 pA, n = 5). These data suggest a direct postsynaptic effect on membrane properties.

## Discussion

The key findings of this study are as follows. Synaptic transmission and neuronal excitability in SG neurons are increased in slices from arthritic rats compared to control neurons from normal rats. These data suggest plastic changes in the arthritis pain model that are maintained in the reduced slice preparation independently of peripheral and supraspinal influences. Blockade of CGRP receptors inhibits synaptic plasticity in SG neurons from arthritic animals, suggesting the contribution of endogenously activated CGRP receptors. CGRP facilitates synaptic transmission and increases neuronal excitability through a postsynaptic site of action.

This study focused on synaptic transmission of afferent information to SG neurons. According to conventional criteria such as stable latencies of the EPSC peak [48,49], synaptic responses were considered monosynaptic and had a latency that indicated a slow conduction velocity (CV) of the responsible afferents in the range of rodent C-fibers [48,49,52]. However, a note of caution should be added. Since we were not able to preserve long dorsal roots in the majority of the experiments but rather stimulated the DR stump near the DREZ, we can not be sure that the calculated CV at the central terminal accurately reflects the CV in the axon of the peripheral fiber. Still, it is evident that afferent input to SG neurons from small diameter fibers but not fast conducting A-beta fibers was studied here. Further, the SG neurons included in this study fulfilled the criteria for central SG neurons with monosynaptic C-fiber input as described in detail by others [48,49].

Our data show for the first time changes of synaptic transmission in small diameter fibers to SG neurons in a model of arthritic pain. Previously, the slice preparation had been used to determine changes in transmission to SC neurons in an inflammatory pain model induced by intraplantar complete Freund's adjuvant 48 h or 7–10 d before slices were obtained [4,5,9]. In these studies, changes in synaptic transmission had been observed. They included a lower threshold for evoking EPSCs in SG neurons, a relative increase in the C-fiber versus A-delta fiber evoked EPSC amplitude, and an increased percentage of SG neurons receiving mono- or polysynaptic A-beta input [4,5,9].

The present study extends these observations in several ways. We used a different pain model, the K/C induced arthritis that produces electrophysiological and behavioral changes in vivo with a well defined and highly reproducible time course [10,11,16,17]. This allowed us to select a constant time point (6 h postinduction of arthritis) to study changes in the slice preparation. Arthritis pain-related changes reach a plateau 6 h postinduction and persist at that level for days. Another novel aspect of our studies is the analysis of complete input-output functions of the DR-SG synapse and of neuronal excitability of SG neurons. Our data show synaptic plasticity combined with excitability changes in the arthritis pain model. There was no significant change of the threshold for evoking EPSCs suggesting that the stimulation and recording conditions were indeed comparable in slices from normal and arthritic animals. The fact that differences of synaptic transmission between normal and arthritic conditions were observed at different stimulus intensities along the input-output relationships further suggests functional changes rather than variability of the experimental conditions.

The present study also offers a mechanism for these plastic changes: enhanced function of CGRP1 receptors. A widely used selective CGRP1 receptor antagonist (CGRP8-37) [18,20,21] inhibited synaptic plasticity but had little effect on normal transmission. These findings are consistent with in vivo data showing that blockade of spinal CGRP receptors inhibits sensitization of dorsal horn neurons in pain models [28,29] and nociceptive behavior [25,25,41-45] in pain models. Here we show for the first time that the DR-SG circuitry is one site of action of CGRP receptors to modulate synaptic transmission. CGRP containing terminals and CGRP receptors are present in the dorsal horn, including SG [30-33]. CGRP is released in the dorsal horn in the K/C arthritis model [34].

Our data with exogenously administered CGRP further indicate a post- rather than pre-synaptic site of action at the DR-SG synapse and a direct membrane effect on SG neurons. This mechanism of action could explain the CGRP-induced sensitization of dorsal horn neurons in vivo [23,28,29,38,39,39] and facilitation of nociceptive behavior [24,37,38]. CGRP has been shown before to depolarize and increase excitability of dorsal horn neurons in current-clamp [40]. Our simultaneous recording and analysis of evoked synaptic transmission, miniature EPSCs and membrane currents in voltage-clamp and excitability in current-clamp show a direct facilitatory action of CGRP on SG neurons to increase their responsiveness to afferent input and their output (action potential generation). The enhanced CGRP function in the arthritis pain model could involve a change in the coupling to downstream effector systems such as kinases and ion channels as well as increased receptor expression or affinity. The effects of peripheral inflammation on CGRP binding sites in the dorsal horn have been reported to be somewhat inconsistent in that a mixture of up- and downregulation was found [32,36]. The inhibitory effect of CGRP on normal synaptic transmission observed in some neurons could reflect an action on the recently described inhibitory projection islet cells on central SG neurons [48].

SG neurons with C-fiber input (such as those selected in the present study) have been shown to excite monosynaptically SG neurons with A-delta input. These neurons then excite monosynaptically lamina I neurons [49], some of which project rostrally to form the spino-parabrachio-amygdaloid pathway [49,53,54]. This pathway is highly peptidergic and utilizes CGRP to transmit information to the amygdala [10]

## Conclusion

This study is the first to show synaptic plasticity in the spinal dorsal horn (SG) in a model of arthritic pain. Synaptic plasticity involves CGRP1 receptor activation. CGRP acts postsynaptically to increase the input and output functions of SG neurons.

## Methods

Male Sprague Dawley rats (16–21 d) were housed in a temperature controlled room and maintained on a 12 h day/night cycle. Water and food were available ad libitum. Electrophysiological data were obtained from untreated normal rats and rats with monoarthritis in the knee (6 h after induction). All experimental procedures were approved by the Institutional Animal Care and Use Committee (IACUC) at the University of Texas Medical Branch (UTMB) and conform to the guidelines of the International Association for the Study of Pain (IASP) and of the National Institutes of Health (NIH).

### Arthritis pain model

In the group of arthritic rats, arthritis was induced in the left knee joint as previously described [7,10,11]. A kaolin suspension (4%, 100 μl) was injected into the left knee joint cavity through the patellar ligament. After repetitive flexions and extensions of the knee for 15 minutes, a carrageenan solution (2%, 100 μl) was injected into the knee joint cavity, and the leg was flexed and extended for another 5 minutes. Spinal cord slices were obtained 6 h after arthritis induction.

### Spinal cord slice preparation

Transverse spinal cord slices were prepared using a modified version of the technique established by E.R. Perl's group [48,49]. Rats were deeply anesthetized with pentobarbital (50 mg/kg, i.p.). After a lumbosacral laminectomy the spinal cord with associated dorsal roots on one side was quickly removed and placed in ice-cold, sucrose-substituted, artificial cerebrospinal fluid (sucrose ACSF) containing (in mM): sucrose (234), KCl (3.6), CaCl_2 _(2.5), MgCl_2 _(1.2), NaH_2_PO_5 _(1.2), NaHCO_3 _(25), and glucose (12); equilibrated to pH 7.4 with a mixture of 95% O_2 _and 5% CO_2_. A vibrotome (Camden Instruments, London, UK) was used to prepare transverse (500 μm thick) slices from the lumbar spinal cord. Spinal cord slices were maintained at room temperature (21°C) for at least 1 h in standard ACSF containing (in mM): NaCl (117), KCl (4.7), NaH_2_PO_4 _(1.2), CaCl_2 _(2.5), MgCl_2 _(1.2), NaHCO3 (25), glucose (11); equilibrated to pH 7.4 with 95% O_2 _/5% CO_2_. A single slice was then transferred to the recording chamber and submerged in ACSF, which superfused the slice at ~5 ml/min.

### Electrophysiology

Whole-cell current- and voltage-clamp recordings were made from substantia gelatinosa (SG) neurons in transverse lumbar spinal cord slices (500 μm) from normal rats (controls) and arthritic rats (16–21 day old), using DIC-enhanced infrared video-microscopy for visualization or the "blind" patch technique as in our previous studies [7,10,11]. Patch electrodes were made from borosilicate glass capillaries (1.5 mm outer diameter, 1.12 mm inner diameter; Drummond, Broomall, PA) pulled on a Flaming-Brown micropipette puller (P-80/PC; Sutter Instruments, Novato, CA). Patch electrodes had tip resistances of 4–6 MΩ. The following internal solution was used (compounds in mM): K-Gluconate (122), NaCl (5), CaCl_2 _(0.3), EGTA (1), HEPES (10), Na_2_ATP (5), Na_3_GTP (0.4), and MgCl_2_(2); pH 7.3; 300 mOsm.

Recording electrodes were positioned in the center of the SG under visual control. The boundaries of the SG are easily discerned under light microscopy. After tight (> 1 GΩ) seals were formed and the whole-cell configuration was obtained, neurons were included in the sample if the resting membrane potential was more negative than -50 mV and action potentials overshooting 0 mV were evoked by direct depolarizing current injection through the recording electrode. Data acquisition and analysis of voltage and current signals were done using a dual 4-pole Bessel filter (Warner Instrument Corp., Hamden, CT), low-noise Digidata 1322 interface (Axon Instruments, Foster City, CA), Axoclamp-2B or Axopatch 200 B amplifiers (Axon Instr.), Pentium PC, and pCLAMP8 and pCLAMP9 software (Axon Inst.). Recordings were made at -60 mV. Series resistance was at least one order of magnitude less than input resistance and was continuously monitored throughout the experiment.

Monosynaptic excitatory postsynaptic currents (EPSCs) were evoked by stimulation of the DR with a suction electrode or, in the majority of experiments, by focal stimulation of the DR stump near the DREZ with a concentric bipolar electrode. Electrical stimuli (150 μs square-wave pulses) were delivered at frequencies below 0.25 Hz. Input-output relationships were obtained by increasing the stimulus intensity in 50 or 100 μA steps. For the evaluation of a drug effect on synaptically evoked responses, the stimulus intensity was adjusted to 80% of the intensity required for orthodromic spike generation. EPSCs were judged to be monosynaptic on the basis of stable latencies of the EPSC peak amplitude (coefficient of variation < 2% [48,49]). CV was estimated from latency of the evoked EPSC peak and the conduction distance between stimulation and recording sites.

The following parameters were recorded to measure arthritis-related or drug-induced changes. Peak amplitude and area under the curve (AUC) of monosynaptic EPSCs (typically the mean of 8–10 consecutive EPSCs) were measured in voltage-clamp to determine synaptic strength and total charge, respectively. Frequency and amplitude of miniature EPSCs (mEPSCs, recorded in 1 μM TTX) were determined from 1 min recording periods (voltage-clamp) using the Mini Analysis Program 6.0.3 (Synaptosoft Inc., Decatur, GA). Input-out functions of excitability were calculated from the number of evoked action potentials (spikes) evoked by direct intracellular injections of depolarizing currents (500 ms; increments of 50 pA).

### Drugs

CGRP (receptor agonist) and CGRP8-37 (selective CGRP1 receptor antagonist) [18,20,21] were dissolved in ACSF on the day of the experiment and applied to the spinal cord slice by gravity-driven superfusion in the ACSF for 10 min at a rate of 5 ml/min. Solution flow into the recording chamber (1 ml volume) was controlled with a three-way stopcock. Duration of drug application and concentrations were selected based on our previous studies [10].

### Data analysis and statistics

All averaged values are given as the mean ± SEM. Statistical significance was accepted at the level of P < 0.05. Input-output functions and concentration-response relationships were compared using a two-way ANOVA with Bonferroni posttests. EC_50 _values were calculated from sigmoid curves fitted to the cumulative concentration-response data by nonlinear regression using the formula *y *= *A *+ (*B *- *A*)/[1 + (10^*C*^/10^*X*^)^*D*^], where A = bottom plateau, B = top plateau, C = log(IC_50_), D = slope coefficient (GraphPad Prism 3.0). Resting transmembrane potentials (RMP), input resistance and spike threshold of neurons from normal rats and from arthritic rats were compared using an unpaired t-test. Drug effects on EPSC peak and AUC, spike threshold, and mEPSC amplitude and frequency were compared to predrug control values using the paired t-test. mEPSCs frequency and amplitude distributions were determined using the Mini Analysis Program 6.0.3 program (Synaptosoft Inc.). The Kolmogorov-Smirnov test was used for statistical analysis of the cumulative distribution of mEPSC amplitude and frequency.

## Abbreviations

CeA, central nucleus of the amygdala

CGRP, calcitonin gene-related peptide

CNS, central nervous system

CV, conduction velocity

DR, dorsal root

DREZ, dorsal root entry zone

EPSC, excitatory postsynaptic current

K/C, kaolin and carrageenan

Ri, input resistance

RMP, resting transmembrane potential

SG, substantia gelatinosa

## Competing interests

The author(s) declare that they have no competing interests.

## Authors' contributions

GCB carried out the majority of the experiments. VN, JSH and HA performed additional experiments. GCB, JSH, YF, HA and VN performed the data analysis. VN and WDW conceptualized the project and formulated the hypothesis. VN designed and directed the experiments and wrote the manuscript.
